# Evaluation of bilateral central retinal artery occlusions with optical coherence tomography-based microangiography: a case report

**DOI:** 10.1186/s13256-016-1095-0

**Published:** 2016-11-01

**Authors:** Aaron Y. Lee, Qinqin Zhang, Douglas M. Baughman, Raghu Mudumbai, Ruikang K. Wang, Cecilia S. Lee

**Affiliations:** 1 Department of Ophthalmology, University of Washington, Seattle, WA USA; 2 Department of Bioengineering, University of Washington, Seattle, WA USA

**Keywords:** Central retinal artery occlusion, Optical coherence tomography angiography (OCTA), Optical coherence tomography-based microangiography (OMAG), Case report

## Abstract

**Background:**

We report a case of bilateral central retinal artery occlusion and the evaluation of retinal vasculature and capillaries by using optical coherence tomography angiography.

**Case presentation:**

A 75-year-old white man presented with central retinal artery occlusion in one eye and underwent a carotid angioplasty. Upon discontinuing anticoagulant, he had a subsequent central retinal artery occlusion in the other eye. Optical coherence tomography angiography images were obtained to compare the retinal microvasculature in both eyes.

**Conclusions:**

Atrophy of the involved retina continues for several weeks after central retinal artery occlusion but the loss of retinal capillaries is immediate and stable over time. The presence of cilioretinal arteries that perfuse the central macula can prevent profound vision loss.

## Background

Central retinal artery occlusion (CRAO) is an important vascular cause of serious vision loss. The incidence of non-arteritic CRAO is estimated to be 1 in 100,000 people [1] and most result in profound vision loss [2]. Despite being a well-recognized ophthalmic emergency [3], limited progress has been made in diagnostic evaluations. Studies suggest that CRAO lasting approximately 240 minutes results in irreparable retinal damage [3, 4].

Fluorescein angiography (FA) has been a standard method in assessing retinal vasculature and diseases such as CRAO [5]. Sodium fluorescein is given intravenously and the fluorescence emitted from retinal and choroidal circulation is detected on angiography. CRAO can be diagnosed clinically with common funduscopic findings such as diffuse retinal edema, “cherry-red spot,” “box-carring,” or attenuation of the vessels [6, 7]. The delayed filling of the affected vessels on FA confirms the diagnosis. However, FA is invasive and relatively contraindicated in pregnancy or chronic kidney failure. In addition, the visualizations of retinal vasculature are limited with this imaging [5]. Recently, the introduction of optical coherence tomography angiography (OCTA) has shown promising results in allowing noninvasive functional imaging of retinal vasculature [8]. With this imaging modality, both structural and functional changes of the retina can be assessed.

Optical coherence tomography-based microangiography (OMAG) is an OCTA algorithm and has been used for evaluation of various retinal diseases [9, 10]. With this technology, the retinal microvasculature can be visualized, quantified, and dissected in various layers. There has been increasing interest in evaluating the area of retinal ischemia following retinal arterial occlusions using structural images of spectral domain OCT (SD-OCT) without the angiography component [5, 11]. Using OMAG, the superficial, intermediate, and deeper retinal capillary plexus can be visualized *in vivo* in addition to structural images. The purpose of this case report is to describe key retinal structure changes and vasculature findings in a case of bilateral CRAOs using fundus photo, SD-OCT, and OMAG.

## Case presentation

### Clinical details

**Fig. 1:**
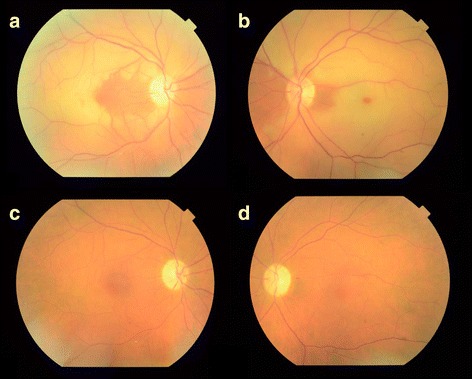
Fundus color photographs of both eyes. **a** One week after foveal-sparing central retinal artery occlusion in the right eye, diffuse macular edema is present except for nasal parafovea and perifovea. Hollenhorst plaque is noted. **b** Four days after the central retinal artery occlusion in the left eye, diffuse macular edema is noted with cherry red spot. The nasal and temporal peripapillary retina are unaffected. **c** Three months after the central retinal artery occlusion in the right there is attenuation of the arter﻿ioles. **d** Three months after the central retinal artery occlusion in the left eye

A 75-year-old white man with a history of ischemic cardiomyopathy status post-heart transplant presented with acute loss of vision in his right eye for 1 week, no history of polymyalgia rheumatica, and no scalp tenderness, jaw claudication, proximal muscle weakness, or temporal headache. His best-corrected visual acuity was 20/25 eccentrically in his right eye and 20/25 in his left eye. Fundus imaging showed several Hollenhorst plaques and diffuse whitening of his retina except for the nasal macula (Fig. 1a). FA was not obtained due to severe chronic renal insufficiency.

He underwent a stroke workup including a complete blood count (CBC), erythrocyte sedimentation rate (ESR), C-reactive protein (CRP), carotid Doppler, echocardiogram, and magnetic resonance imaging/angiography (MRI/MRA) of his brain. Of note, his platelet count was 148×10^3^/uL, ESR 21, and CRP was 7.4 mg/L. An approximate 90 % stenosis at the origin of his innominate artery with 30 % stenosis of the right carotid bulb was found. He underwent balloon angioplasty and stenting of his innominate artery and was placed on a daily dose of aspirin (325 mg) and clopidogrel (75 mg); however, he stopped the medicine due to poor tolerance.

Four months after the CRAO in his right eye and a few weeks after discontinuing clopidogrel, he had another episode of acute painless vision loss involving his left, previously unaffected, eye. At this time, his visual acuity was 20/30 eccentrically in his right eye and 20/400 in his left eye. A funduscopic examination 4 days after the episode of his left eye revealed diffuse whitening of his retina with a cherry red spot (Fig. 1b). A repeat workup was unremarkable and he was restarted on clopidogrel. Fundus photographs were repeated three months after the CRAO in each eye (Fig. 1c, d).

### Investigations

He underwent a comprehensive ophthalmologic examination and color fundus imaging (Topcon, Tokyo, Japan) tests and SD-OCT imaging (Heidelberg, Heidelberg, Germany) tests whenever possible during his initial visit and follow-up visits. In addition, OMAG imaging was obtained subsequently using a modified SD-OCT (CIRRUS prototype provided by Carl Zeiss Meditec Inc., Dublin, CA, USA) with a central wavelength of 850 nm and a speed of 100,000 A-scans per second.

The details of OMAG scanning protocol and algorithm have been described previously [9]. The complex version of the OMAG algorithm was applied to the images that were extracted. The retinal images were segmented into three different retinal layers using a semi-automated algorithm: an inner retinal layer from the ganglion cell layer to the inner plexiform layer, a middle retinal layer from the inner nuclear layer to the outer plexiform layer, and an outer retinal layer from the external limiting membrane and the outer nuclear layer. The three-dimensional structure of the retina was projected using Matlab (The MathWorks, Inc., Natick, MA, USA).

The mean retinal thickness was measured by calculating the average of five consecutive scans. The macula was divided into nine zone Early Treatment Diabetic Retinopathy Study (ETDRS) grids: parafovea, inner/outer nasal, inner/outer temporal, inner/outer superior, and inner/outer inferior. Perfusion index was defined as the percent coverage of the area by retinal vessels with flow. For his right eye, the perfusion indices were compared between two subsequent visits following the foveal-sparing CRAO. For his left eye, the perfusion indices before and after CRAO were compared.

**Fig. 2:**
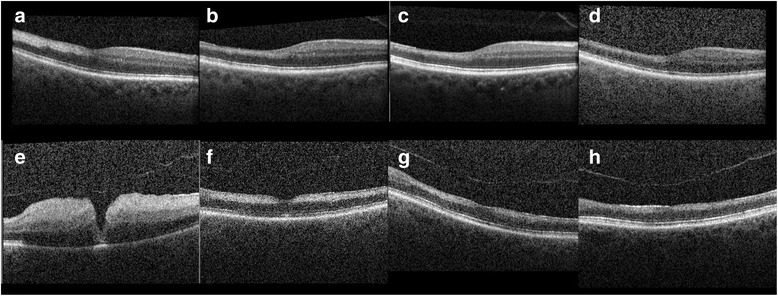
Spectral domain optical coherence tomography of both eyes. **a**–**d** Spectral domain optical coherence tomography of the right eye obtained at 1, 3, 5, and 7 months respectively after the central retinal artery occlusion. **e**–**h**. Spectral domain optical coherence tomography of the left eye obtained 4 days, 1 month, 3 months, and 5 months respectively after the central retinal artery occlusion

**Fig. 3:**
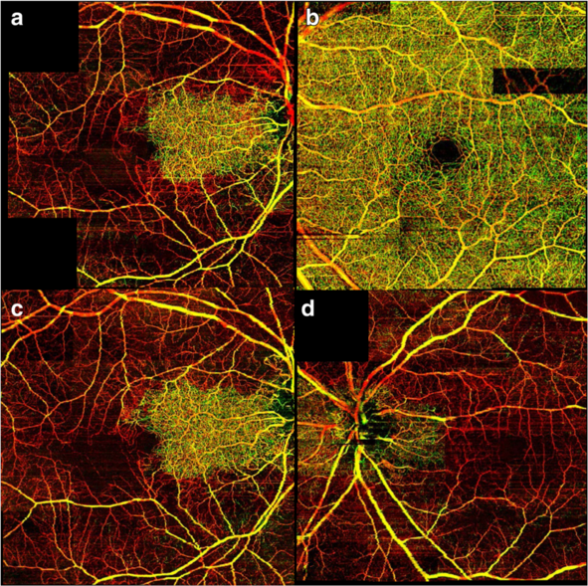
Optical coherence tomography-based microangiography images of both eyes. **a** Enface image obtained at 1 month after central retinal artery occlusion in the right eye. **b** The optical coherence tomography-based microangiography image of the left eye prior to the central retinal artery occlusion in the left eye. **c** ﻿Enface image obtained at 3 months after central retinal artery occlusion in the right eye. No significant difference is shown between the enface images obtained 1 and 3 months after the central retinal artery occlusion in the right eye. **d** One month after the central retinal artery occlusion in the left eye, diffuse loss of microvasculature is shown. Color schemes are based on the anatomical depth with *green* color being the most superficial followed by *yellow* and *red*

SD-OCT imaging was obtained at 1, 3, 5, and 7 months after the foveal-sparing CRAO in his right eye (Fig. 2a–d). Both superficial and deep retinal layer thickness decreased in the involved temporal macula compared to the nasal macula. Mean retinal thickness temporal to fovea (500 μm away from the foveal center) in his right eye at months 1, 3, 5, and 7 was 250 μm, 204 μm, 194 μm, and 191 μm, respectively. OMAG images of his right eye at 1 and 3 months are shown in Fig. 3a, c. OMAG images of his left eye prior to the CRAO and 1 month after the CRAO are shown In Fig. 3b, d.

SD-OCT imaging was obtained on day 4 and at 1 month, 3 months, and 5 months after the CRAO in his left eye. (Fig. 2e–h) On day 4, there was diffuse edema involving both superficial and deep retinal layers. Mean retinal thickness temporal to fovea (660 μm away from the foveal center) was 504 μm. Follow-up images of the same area revealed thicknesses of 246 μm, 215 μm, and 196 μm, at months 1, 3, and 5, respectively.

**Fig. 4:**
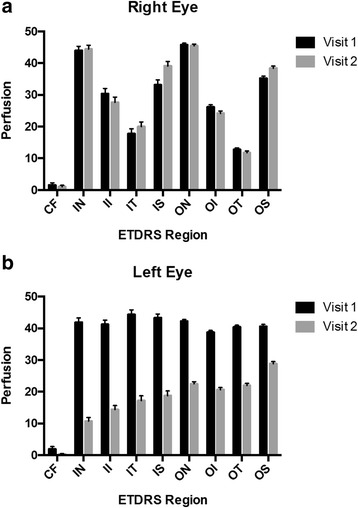
Bar graphs of mean perfusion indices in nine zones of macula with error bars representing standard error. **a** Right eye on visits 1 and 2 following central retinal artery occlusion. **b** Left eye before (visit 1) and after (visit 2) central retinal artery occlusion. *CF* central fovea, *ETDRS* Early Treatment Diabetic Retinopathy Study, *II* inner inferior, *IN* inner nasal, *IS* inner superior, *IT* inner temporal, *OI* outer inferior, *ON* outer nasal, *OS* outer superior, *OT* outer temporal

Perfusion indices showed no significant difference during two consecutive visits after the CRAO in his right eye (Fig. 4). The perfusion indices in all zones were below 40 except for the inner and outer nasal ETDRS zones which were unaffected by the CRAO. There was a statistically significant difference in the perfusion indices of all nine zones before and after the CRAO in his left eye (*p*-value <0.001, Fig. 4). The average perfusion index in the eight non-central zones prior to the CRAO was 40 (range 39 to 45) while the average perfusion index after the CRAO in the non-central zones was 20 (range 10 to 30).

### Outcomes

The retinal vasculature loss following CRAO was acute and permanent, but the retinal atrophy progressed over several months. In our patient, the thickness of inner retina continued to decrease until month 5, but then it stabilized on follow-up visits. However, the structural architecture of nasal retina remained undisrupted by SD-OCT throughout the follow-up period. OMAG images showed severe loss of all retinal microvasculature involving intermediate and deep plexus more than the superficial plexus. The en face OMAG images obtained at months 1 and 3 demonstrated visible absence of the retinal microvasculature in his right eye except for the nasal macula (Fig. 3a, c). The en face OMAG image obtained at month 1 after the CRAO in the left eye revealed loss of retinal capillary plexus in the entire macula (Fig. 3d).

Even though the FA is a standard imaging modality in evaluating retinal vascular diseases, FA is often not possible due to time commitment and patient factors such as renal insufficiency. Although FA would have been diagnostic in our patient, it was deferred due to chronic renal insufficiency. Contraindications for FA include fluorescein allergy, renal failure, pregnancy, moderate-severe asthma, and significant cardiac cases [12]. The noninvasive nature of OCTA is advantageous in many of our patients with multiple comorbidities. In addition, the image quality can vary due to media opacity such as cataract and patient cooperation. Smaller capillaries and deeper retinal plexus are not visible with FA. In contrast, OCTA provides a safe and potentially superior alternative method of assessing total retinal vasculature.

Recently, retinal artery occlusions were characterized as superficial and/or intermediate and deep capillary plexus ischemia using SD-OCT images [11]. In particular, the ischemia within the intermediate and deeper retinal capillary plexus were described as a hyper-reflective lesion at the level of the inner nuclear layer and referred to as paracentral acute middle maculopathy [13]. The characteristic ischemia in the inner retina cannot be presumed based only on the change in the reflective pattern seen on SD-OCT, but OCTA allows for direct visualization of perfusion and quantification. Thus, the vascular change evident on OCTA such as OMAG could become an important clinical endpoint in future studies.

Our patient had extensive involvement of all three retinal capillary plexuses. In particular, the loss of vasculature in the deep capillary plexus appeared more profound in the ischemic areas compared to the perfused nasal retina supplied by the cilioretinal artery in both eyes. Unfortunately, the cilioretinal artery of our patient’s left eye did not supply the fovea, thus he had more profound vision loss in this eye. The presence of cilioretinal arteries that contribute to the macula has been previously reported in 18.7 % of patients [14], but there is high variability in the number, vessel caliber, and location of cilioretinal arteries in the general public.

Perfusion indices are potentially objective values that can be followed over time. These values can be interpreted as the areas of significant flow and low numbers would indicate either decreased flow or area of ischemia. In our analysis, the perfusion index was calculated using the perfusion percentage within independently subsampled areas of the retina. This method allowed the quantification of the OCTA signal per each ETDRS zone leading to a more robust quantification.

To the best of our knowledge, this is the first description of OCTA imaging in bilateral CRAO and comparison of the OCTA imaging before and after CRAO. OCTA provides important information regarding vascular changes including hypoperfusion, ischemia, and abnormal structure. However, the lack of standardizations, the lack of full understanding of the artifacts, and the lack of large trials to determine the clinical relevance of image findings are the main current limitations. Despite these challenges, OCTA such as OMAG will probably become an effective adjunct modality in diagnosing and following retinal vascular diseases.

## Conclusions

CRAO is a relatively common sight-threatening condition. Using OCTA, we demonstrate that the atrophy of the involved retina continues for several weeks following CRAO but the loss of retinal capillaries is immediate and stable over time. The presence of cilioretinal arteries that perfuse the central macula can prevent profound vision loss.

## Abbreviations

CBC: Complete blood count
CRAO: Central retinal artery occlusion
CRP: C-reactive protein
ESR: Erythrocyte sedimentation rate
ETDRS: Early Treatment Diabetic Retinopathy Study
FA: Fluorescein angiography
MRI/MRA: Magnetic resonance imaging/angiography
OCTA: Optical coherence tomography angiography
OMAG: Optical coherence tomography-based microangiography
SD-OCT: Spectral domain optical coherence tomography
